# Exploratory immunogenicity outcomes of peanut oral immunotherapy: Findings from the PALISADE trial

**DOI:** 10.1002/clt2.12326

**Published:** 2024-01-17

**Authors:** Caroline Nilsson, Andrea Vereda, Magnus P. Borres, Mats Andersson, Eva Södergren, Magnus Rudengren, Alex Smith, Reyna J. Simon, Robert Ryan, Montserrat Fernández‐Rivas, Daniel Adelman, Brian P. Vickery

**Affiliations:** ^1^ Clinical Research and Education Karolinska Institutet Sachs' Children and Youth Hospital Stockholm Sweden; ^2^ Aimmune Therapeutics, a Nestlé Health Science Company London UK; ^3^ Karolinska University Hospital Stockholm Sweden; ^4^ Thermo Fisher Scientific Uppsala Sweden; ^5^ Aimmune Therapeutics, a Nestlé Health Science Company Brisbane California USA; ^6^ Calico Life Sciences South San Francisco California USA; ^7^ Allergy Department Hospital Clínico San Carlos Madrid Spain; ^8^ Department of Medicine University of California‐San Francisco San Francisco California USA; ^9^ Emory University School of Medicine‐Pediatrics Atlanta Georgia USA

**Keywords:** immunoglobulin, oral immunotherapy, peanut, peanut allergy, serology

## Abstract

**Background:**

Immunoglobulin E (IgE) and immunoglobulin G4 (IgG4) to peanut and its components may influence the clinical reactivity to peanut. Allergen‐specific immunotherapy is known for modifying both IgE and IgG4. Peanut oral immunotherapy may influence these serological parameters.

**Methods:**

Exploratory analyses of serological data from participants receiving peanut (*Arachis hypogaea*) allergen powder‐dnfp (PTAH) and placebo in the double‐blind, randomized, phase 3 PALISADE trial were conducted to evaluate potential relationships between peanut‐specific and peanut component–specific (Ara h 1, Ara h 2, Ara h 3, Ara h 6, Ara h 8, and Ara h 9) IgE and IgG4 levels and clinical outcomes.

**Results:**

A total of 269 participants (PTAH, *n* = 202; placebo, *n* = 67) were analyzed. No relationship was observed between specific IgE and IgG4 levels at screening and maximum tolerated peanut protein dose during screening or response status during exit double‐blind placebo‐controlled food challenge (DBPCFC). In PTAH‐treated participants, no relationship was observed between IgE and IgG4 levels at screening and maximum symptom severity during exit DBPCFC. Postscreening ratios (ie, postscreening/screening) in the PTAH group were significant at the end of updosing and exit visit for most components. Postscreening changes in specific IgE levels were more pronounced with PTAH versus placebo for most components.

**Conclusions:**

Specific IgE and IgG4 levels at screening are not correlated with screening or exit DBPCFC results, and are not predictive of clinical response to PTAH. Peanut (*Arachis hypogaea*) allergen powder‐dnfp contains the relevant and immunodominant allergens, inducing immunological changes with the treatment.

**Clinical Trial Registration:**

ClinicalTrials.gov identifier: NCT02635776.

## 1 INTRODUCTION

The prevalence of peanut allergy is currently estimated at 2%–3% in infants and children^1^, ^2^, ^3^ and is rising in the industrialized world.^4^ In some affected individuals, potential allergic responses may include anaphylaxis, even with exposure to milligram quantities of peanut protein.^5^ The allergy is usually long‐lasting (ie, into adulthood).^6^ Clinical presentation of peanut allergy may be influenced by the presence, characteristics, or relationships between various antibodies (specific Immunoglobulin E (IgE) and blocking immunoglobulin G4 (IgG4)).^7^, ^8^ Clinical reactivity has also been correlated with IgE epitope patterns.^7^

Until now, the standard of care for peanut allergic patients solely included educating families about the risks of exposure, coaching them on avoidance strategies, and training them to manage acute allergic reactions. Some patients may now complete their treatment strategy with controlled exposure using an approved and regulated peanut oral immunotherapy (OIT) in children to achieve desensitization that could protect them from accidental exposures.^9^, ^10^ Following immunotherapy for peanut allergy, allergen‐specific IgG4 levels have been found to increase^11^, ^12^, ^13^, ^14^ and specific IgE levels to peanut and peanut skin prick test to be reduced,^11^, ^12^, ^14^ which is aligned with serology changes observed with the rest of the allergen‐specific immunotherapies.^15^, ^16^, ^17^, ^18^, ^19^

Peanut (*Arachis hypogaea*) allergen powder‐dnfp (PTAH; PALFORZIA^®^, previously known as AR101) is a biological product for peanut OIT approved in the United States for the mitigation of allergic reactions, including anaphylaxis, that may occur following accidental exposure to peanut in patients with a confirmed diagnosis of peanut allergy, and in Europe for the treatment of patients with a confirmed diagnosis of peanut allergy.^20^, ^21^ Dose escalation with PTAH may be initially administered to patients aged 4–17 years, and treatment can be continued thereafter, including in older (aged ≥18 years) patients.^20^, ^21^ PTAH demonstrated efficacy and safety in clinical trials, including a phase 3, double‐blind, placebo‐controlled study in children and adults with peanut allergy (PALISADE; NCT02635776).^22^, ^23^, ^24^, ^25^ In PALISADE, changes were noted in peanut‐specific IgE and IgG4 levels from screening to the end of the study in participants treated with PTAH.^23^

The present exploratory analyses of data from PALISADE were conducted to evaluate potential relationships between peanut‐specific and peanut component–specific (Ara h 1, Ara h 2, Ara h 3, Ara h 6, Ara h 8, and Ara h 9) IgE and IgG4 levels and clinical outcomes. Specifically, we aimed to better understand how baseline sensitization patterns predicted the maximum tolerated peanut protein dose or response status and severity of symptoms during food challenges, the effect of PTAH on IgE and IgG4 levels, and the change in IgE and IgG4 levels depending on the response to PTAH.

## 2 METHODS

### 2.1 Trial design and participants

PALISADE was a double‐blind, randomized, placebo‐controlled, multicenter (66 sites in 10 countries in North America and Europe) phase 3 trial.^23^ Details of the study design (summarized schematically in Supplementary Figure S1) were reported previously.^23^ Briefly, eligible participants were aged 4–55 years with a clinical history of peanut allergy, serum IgE to peanut ≥0.35 kU_A_/L and/or skin prick test to peanut ≥3 mm versus control, and dose‐limiting symptoms (DLS) to a ≤100‐mg challenge dose of peanut protein in the screening double‐blind, placebo‐controlled food challenge (DBPCFC) (challenge doses of peanut protein: 1 mg, 3 mg, 10 mg, 30 mg, and 100 mg). Participants reacting during the screening DBPCFC were randomized to PTAH or placebo. Treatment phases included a 1‐day initial dose‐escalation phase (0.5–6 mg PTAH or placebo), an updosing phase (3–300 mg PTAH or placebo; approximately 6 months), and a maintenance phase (300 mg PTAH or placebo; approximately 6 months). The exit DBPCFC (challenge doses of peanut protein: 3 mg, 10 mg, 30 mg, 100 mg, 300 mg, 600 mg, and 1000 mg) was performed after approximately 1 year of total treatment.

The prespecified and post hoc exploratory serological analyses reported here were limited to a subpopulation of participants in PALISADE who received ≥1 dose of study medication, reconsented to have their sera additionally analyzed, and were from the US or United Kingdom (UK). Due to the General Data Protection Regulation (GDPR), sera from participants from other EU countries participating in PALISADE were not included in these analyses. The PALISADE trial was approved by the appropriate ethics committee or institutional review board in each country or participating site and was registered in the European Union Clinical Trials Register (EudraCT Number 2015‐004257‐41) and US National Library of Medicine Clinical Trials database (NCT02635776).

### 2.2 Objectives and assessments

Analyses were conducted to evaluate 1) if the screening sensitization profile was predictive of the maximum tolerated peanut protein dose during the screening DBPCFC; 2) if the screening sensitization profile would predict response status (ie, being a treatment “responder” at the exit DBPCFC to 300 mg, 600 mg, and 1000 mg peanut protein doses); 3) if the screening sensitization profile was predictive of the severity of the symptoms during the exit DBPCFC; 4) the effect of PTAH immunotherapy on specific IgE and IgG4 levels; and 5) the change from screening in specific IgE and IgG4 levels by response status (ie, in treatment “responders” compared with “nonresponders”).

“Responders” were defined as participants tolerating the specified peanut protein dose during the exit DBPCFC without DLS. “Nonresponders” were defined as participants either not tolerating the specified peanut protein dose or those without exit DBPCFC data (ie, those who discontinued early). Single peanut protein doses for evaluating a participant as a responder or nonresponder were the 300 mg, 600 mg, and 1000 mg dose levels.

Blood samples were collected for serum immunoglobulin measurement of peanut‐specific and peanut component–specific (Ara h 1, Ara h 2, Ara h 3, Ara h 6, Ara h 8, and Ara h 9) IgE and IgG4 at screening, the end of updosing, and trial exit (after approximately 12 months of treatment). Sera were stored at −18°C. All samples were analyzed using the ImmunoCAP^®^ technology (Thermo Fisher Scientific, Uppsala, Sweden), using commercially available peanut whole allergen extract and allergen components. Levels of allergen‐specific IgE and IgG4 were reported to a lower threshold of 0.1 kU_A_/L and 0.3 mg_A_/L, respectively.

Symptoms occurring during the DBPCFC were graded as mild, moderate, or severe, consistent with modified PRACTALL consensus guidelines and the Consortium of Food Allergy Research grading system.^26^, ^27^ DLS were symptoms that indicated poor tolerability of the administered challenge dose and precluded safe advance to the next challenge dose per investigator assessment. Mild symptoms requiring pharmacological treatment were also considered DLS.

### 2.3 Statistical analysis

Antibody data (ie, levels of peanut‐specific IgE and IgG4 and all individual components [Ara h 1, Ara h 2, Ara h 3, Ara h 6, Ara h 8, and Ara h 9]) at each visit (screening, end of updosing, and exit) were presented by randomized treatment as geometric means with 95% confidence intervals. Antibody data at screening were presented by maximum tolerated dose at screening DBPCFC, tolerated peanut protein (ie, response status) at exit DBPCFC (separately for 300 mg, 600 mg, and 1000 mg), and maximum symptom severity at exit DBPCFC using box plots.

Changes from screening in peanut‐specific IgE and IgG4 and individual components were independently analyzed with a repeated measures model that had a log of concentration ratio (postscreening/screening) as response and randomized treatment, visit (end of updosing and exit), log of screening (concentration level), treatment by visit, and log of screening by visit interaction as independent factors. The model assumed an unstructured correlation over time and that data were missing at random. From the model, estimates of change from screening by treatment and treatment comparisons were obtained for each postscreening visit and presented as geometric mean ratios and ratios of geometric mean ratios with 95% confidence intervals. In addition, ratios of postscreening to screening in IgE and IgG4 levels were presented at each postscreening visit (end of updosing and exit) by randomized treatment and tolerated peanut protein concentration during the exit DBPCFC (300 mg, 600 mg, or 1000 mg) using geometric means based on observed cases.

Random forest (RF) analyses were performed to investigate if the screening sensitization profile was predictive of being a responder at the exit DBPCFC (separately for 300 mg, 600 mg, and 1000 mg). Only participants from the PTAH group were used in the model. Because there was an imbalance between the number of responders and nonresponders, the larger of the 2 outcomes was undersampled when building the model to ensure similar emphasis on correctly classifying both outcome levels. As the main purpose was to investigate the prediction capabilities of IgE and IgG4 at screening, no other participant characteristics were added to the model. A separate RF model was run (on all participants) to investigate the relationship between the screening sensitization profile and maximum tolerated peanut protein dose at screening DBPCFC. In analyses of the exit DBPCFC (300 mg, 600 mg, 1000 mg peanut protein doses), all participants were included; participants without exit DBPCFC data were considered to be nonresponders for all 3 responder variables. Similarly, for those participants without exit DBPCFC data, maximum symptom severity for analysis was captured as N/A (not applicable). For the responders, participants were counted in analyses of all peanut protein doses they tolerated (ie, participants who tolerated 1000 mg peanut protein on exit DBPCFC also tolerated 300 mg and 600 mg and were included as responders for those analyses as well). Participants that could not tolerate any dose level in the screening DBPCFC were assigned a maximum tolerated dose of 0.3 mg. Analyses of other variables included all participants with available data at each specific time point (observed cases).

To allow for the calculation of logarithms and geometric means, values < 0.1 kU_A_/L for IgE were set to 0.05 kU_A_/L and values < 0.3 mg_A_/L for IgG4 were set to 0.15 mg_A_/L (ie, half the limit of quantitation in each case). Statistical analyses were performed using R statistical software version 4.1.2 (R Foundation for Statistical Computing). Specifically, repeated measures were performed using library nlme v3.1‐153 and RF with library randomForest v4.7‐1. No adjustment was made for multiple comparisons.

## 3 RESULTS

### 3.1 Participants

**FIGURE 1:**
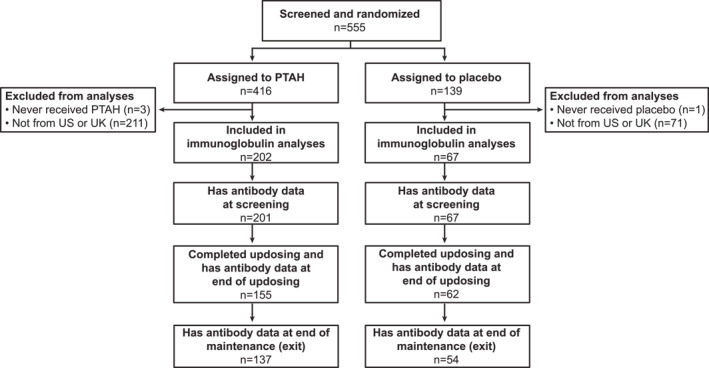
Disposition of participants included in immunoglobulin analyses. PTAH, peanut (*Arachis hypogaea*) allergen powder‐dnfp; UK, United Kingdom; US, United States.

A total of 269 participants were included in the analyses; 202 were treated with PTAH and 67 received placebo (Figure 1). Most (93%) of the participants were recruited in the US. Baseline characteristics, including IgE and IgG4 levels at screening for total peanut and each of its components, are presented in Supplementary Table S1.

### 3.2 Predictability of the maximum tolerated peanut protein dose at screening double‐blind, placebo‐controlled food challenge, based on specific immunoglobulin E and immunoglobulin G4 levels at screening

**FIGURE 2:**
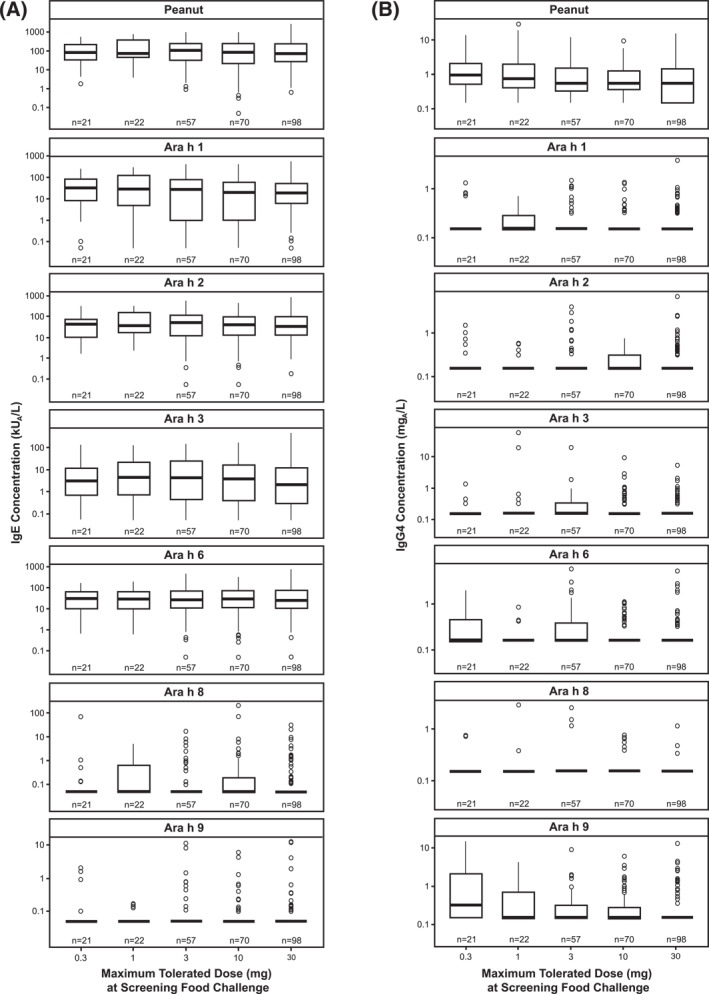
Screening IgE (A) and IgG4 (B) levels by the maximum tolerated peanut protein dose during the screening DBPCFC. DBPCFC, double‐blind, placebo‐controlled food challenge; IgE, immunoglobulin E; IgG4, immunoglobulin G4.

No relationship was observed between specific IgE and IgG4 levels at screening and the maximum tolerated peanut protein dose during the screening DBPCFC (Figure 2). Classification error ranged from 73% (30 mg) to 82% (1 mg) (Supplementary Table S2). The overall error rate was 77%, indicating that only 2 of 10 observations were classified correctly; the maximum tolerated dose was 0.3 mg (*n* = 21), 1 mg (*n* = 22), 3 mg (*n* = 57), 10 mg (*n* = 70), or 30 mg (*n* = 98).

### 3.3 Predictability of response status at exit double‐blind, placebo‐controlled food challenge, based on specific immunoglobulin E and immunoglobulin G4 levels at screening

Responder rates to 300 mg, 600 mg, and 1000 mg of peanut protein doses at exit DBPCFC were 74.8%, 65.8%, and 48.0%, respectively for PTAH; rates were 7.5%, 3.0%, and 1.5% for placebo, respectively (Supplementary Table S3). Exit DBPCFC data were unavailable for 44 PTAH participants and 5 placebo participants, indicating potential early discontinuation from the trial. These participants were included in the nonresponder group. When focusing on the PTAH group included in this analysis, there were no clear differences observed in median specific IgE and IgG4 levels at screening between responders and nonresponders (to the 300 mg, 600 mg, and 1000 mg peanut protein doses at the exit DBPCFC) (Figure 3).

FIGURE 3Screening IgE (A) and IgG4 (B) levels by randomized treatment and response status to the 300 mg, 600 mg, and 1000 mg doses of the exit DBPCFC. Participants at each level of the tolerated dose also tolerated the dose levels below it (ie, those who tolerated 1000 mg of peanut protein at the exit DBPCFC also tolerated 300 mg and 600 mg). DBPCFC, double‐blind, placebo‐controlled food challenge; IgE, immunoglobulin E; IgG4, immunoglobulin G4; PTAH, peanut (*Arachis hypogaea*) allergen powder‐dnfp.

**Figure:**
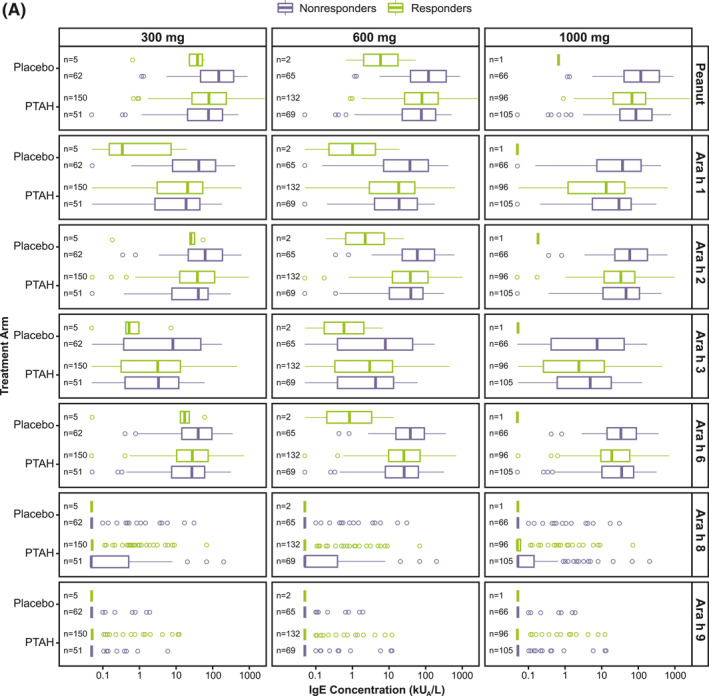

**Figure:**
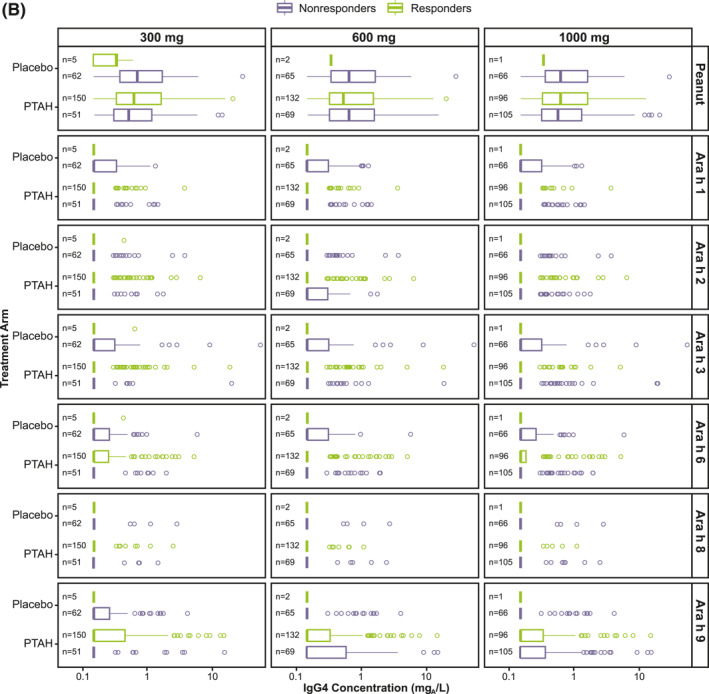

Results of the classification trees (RF) support this finding, since the classification error when trying to predict response status for each participant based on their antibody levels at screening was close to 50% (range 42%–53%) for all 3 response doses (300 mg, 600 mg, and 1000 mg). Supplementary Table S4 shows the observed versus predicted response together with the error rate for each outcome level.

### 3.4 Predictability of maximum symptom severity at exit double‐blind, placebo‐controlled food challenge, based on specific immunoglobulin E and immunoglobulin G4 levels at screening

**FIGURE 4:**
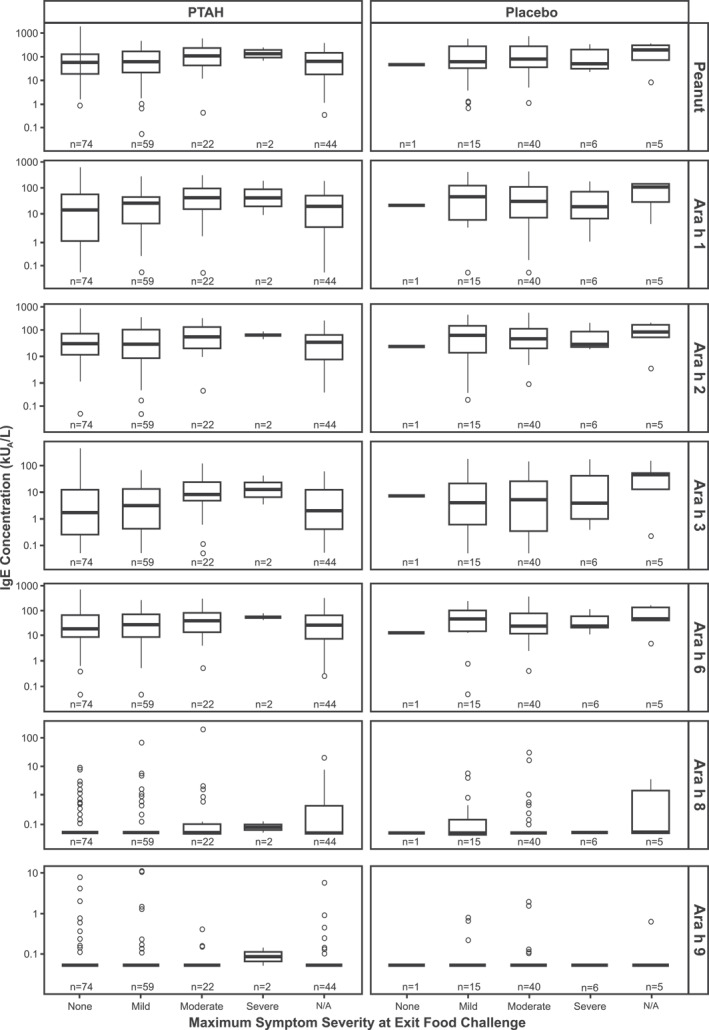
Screening IgE levels by randomized treatment and maximum symptom severity during the exit DBPCFC. N/A corresponds to participants who did not perform the exit DBPCFC. DBPCFC, double‐blind, placebo‐controlled food challenge; IgE, immunoglobulin E; N/A, not applicable.

Although there was no clear relationship observed between IgE and IgG4 levels at screening and maximum symptom severity during the exit DBPCFC, there was a trend between higher screening IgE levels and increasing maximum symptom severity, especially in the PTAH‐treated group (Figure 4 and Supplementary Figure S2).

### 3.5 Effect of immunotherapy on specific immunoglobulin E and immunoglobulin G4 levels

In PTAH‐treated participants, geometric mean component‐specific IgE levels (except for Ara h 8) increased from screening to the end of updosing visit and then decreased at the exit visit (but not to screening levels, including Ara h 9 which reduced by 0.01 kU_A_/L from end of updosing to exit); in placebo‐treated participants, IgE levels remained stable throughout the study period (Figure 5A). Postscreening ratios (ie, postscreening/screening) in the PTAH group were statistically significant at the end of the updosing visit for all components (all *p* < 0.001), except Ara h 8, and at the exit visit for all components (all *p* < 0.05), except Ara h 2 (Figure 5B). In the placebo group, postscreening IgE levels remained stable for all components at both visits (Figure 5B). The changes in specific IgE levels were significantly more pronounced with PTAH compared with placebo (PTAH change/placebo change) at the end of the updosing visit for all components (ratio range, 1.43–2.00) except Ara h 8 (Figure 6A). At the exit visit, only the change in Ara h 9 was significantly higher for PTAH compared with placebo (ratio, 1.39; *p* < 0.01).

FIGURE 5Geometric mean IgE levels (A), the geometric mean change from screening in IgE levels (B), geometric mean immunoglobulin G4 (IgG4) levels (C), and geometric mean change from screening in IgG4 levels (D) by randomized treatment and study visit. *0.01 < *p* ≤ 0.05, **0.001 < *p* ≤ 0.01, ***p ≤ 0.001. *p* values in panels B and D are from repeated measures model. CI, confidence interval; DBPCFC, double‐blind, placebo‐controlled food challenge; IgE, immunoglobulin E; IgG4, immunoglobulin G4; LCL, lower confidence limit; PTAH, peanut (*Arachis hypogaea*) allergen powder‐dnfp; UCL, upper confidence limit.

**Figure:**
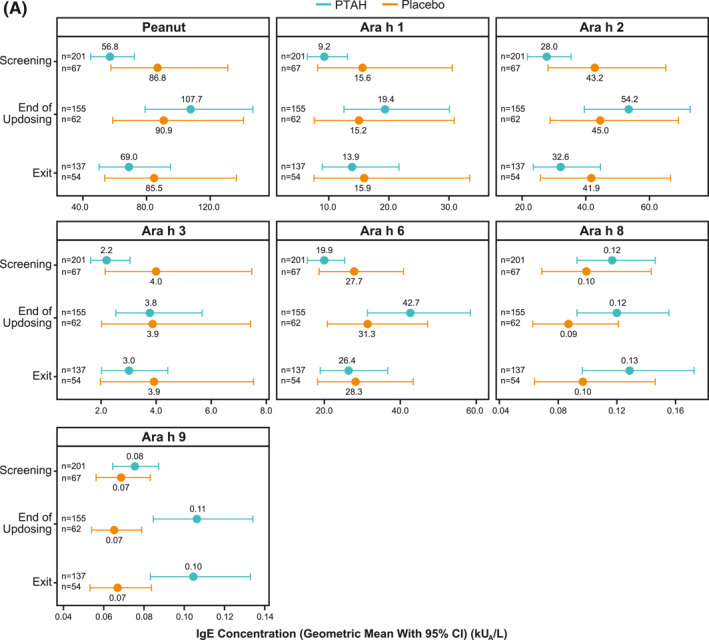

**Figure:**
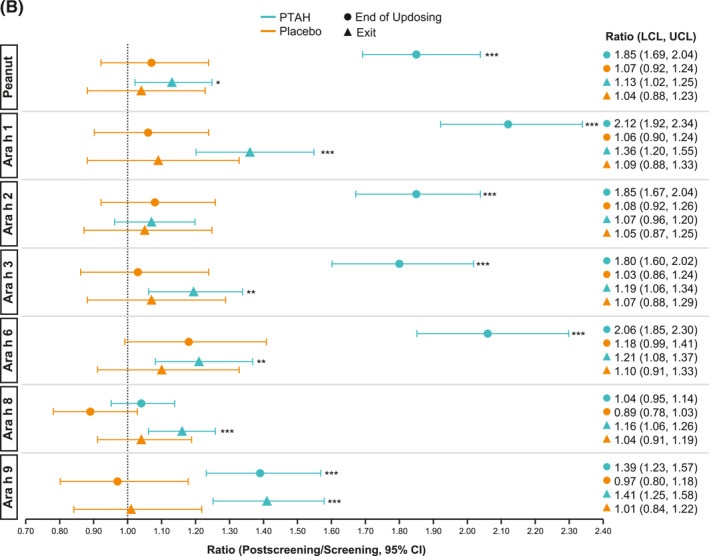

**Figure:**
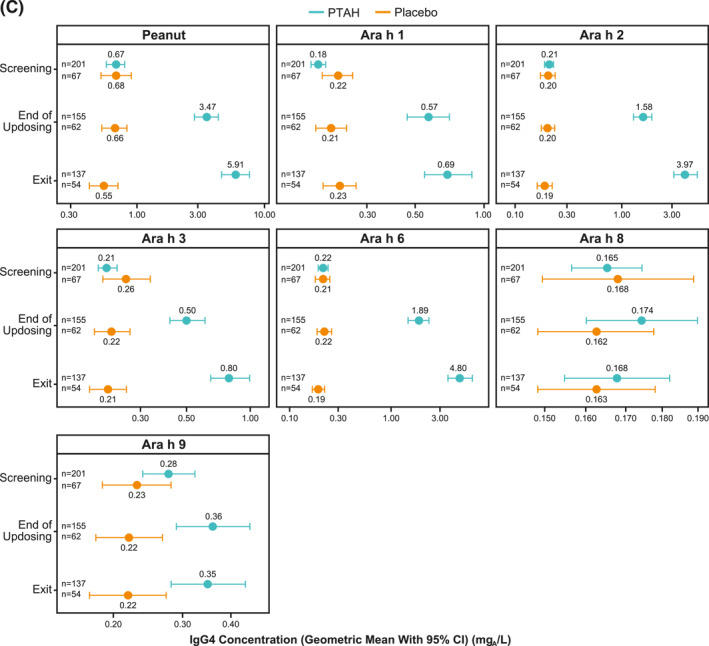

**Figure:**
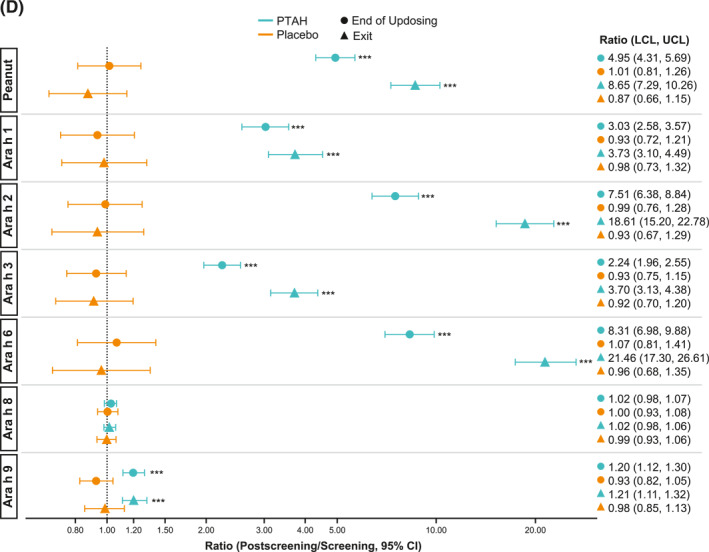

FIGURE 6IgE (A) and IgG4 (B) treatment comparison by study visit (end of updosing and exit). *0.01 < *p* ≤ 0.05, **0.001 < *p* ≤ 0.01, ***p ≤ 0.001. *p* values are from repeated measures model. CI, confidence interval; IgE, immunoglobulin E; IgG4, immunoglobulin G4; PTAH, peanut (*Arachis hypogaea*) allergen powder‐dnfp.

**Figure:**
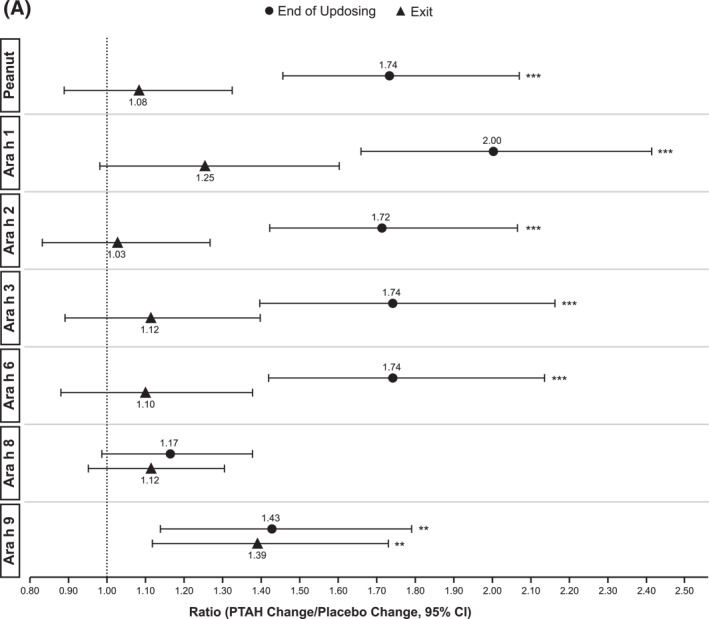

**Figure:**
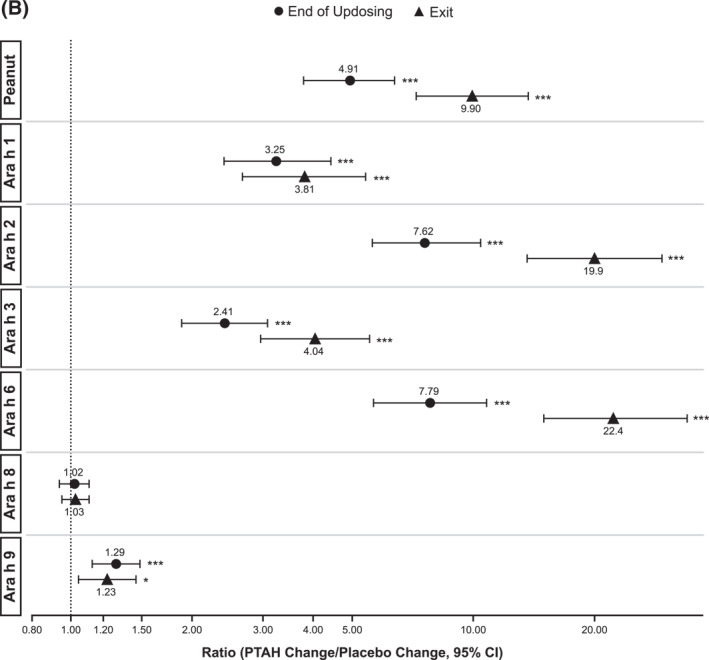

Geometric mean component‐specific IgG4 levels increased during PTAH treatment for all components, except for Ara h 8, whereas Ara h 9–related IgG4 levels were stable between the end of updosing and the exit visit (Figure 5C). In placebo‐treated participants, geometric mean component‐specific IgG4 levels remained stable throughout the study period (Figure 5C). Postscreening IgG4 ratios in the PTAH group were statistically significant for all components (*p* ≤ 0.001), except Ara h 8, at both the end of updosing visit and the exit visit (Figure 5D). In the placebo group, postscreening ratios were not statistically significant at either time point for any peanut component (Figure 5D). Immunoglobulin G4 ratios (PTAH change/placebo change) were significantly higher for PTAH compared with placebo at both visits for all components except Ara h 8 (Figure 6B).

### 3.6 Change of specific immunoglobulin E and immunoglobulin G4 levels from screening between responders and nonresponders

At each peanut protein dose (300 mg, 600 mg, and 1000 mg), and regarding the ratio versus screening of specific IgE, PTAH‐treated responders appeared to have higher values for whole peanut extract and Ara h 1 compared with PTAH‐treated nonresponders (Supplementary Figure S3A). PTAH‐treated responders also appeared to have larger increases in IgG4 levels (whole peanut extract, Ara h 1, Ara h 2, and Ara h 6) than PTAH‐treated nonresponders at peanut protein doses of 300 mg and 600 mg (Supplementary Figure S3B).

## 4 DISCUSSION

In these prespecified and post hoc exploratory analyses, no relationship was observed between specific IgE and IgG4 levels at screening and maximum tolerated peanut protein dose at the screening DBPCFC or the exit DBPCFC (ie, response status). In PTAH‐treated participants, most specific IgE levels increased initially (from screening to the end of updosing) and decreased thereafter (at exit) and most specific IgG4 levels increased during treatment, whereas in placebo‐treated participants, IgE and IgG4 levels remained relatively stable throughout the study period. Changes in most IgE‐and IgG4‐specific components were more pronounced in the PTAH group compared with the placebo group and in PTAH responders compared with PTAH nonresponders.

Other studies of immunologic markers (specific IgE, IgG4, IgE/IgG4, and others) in peanut OIT have been performed, examining issues including screening sensitization as a diagnostic biomarker or predictor of clinical response.^7^, ^8^, ^28^, ^29^ In the phase 2 trial of PTAH, all 6 PTAH‐treated participants who discontinued prematurely (4 due to adverse events, 1 to consent withdrawal, and 1 per investigator decision) had high (>100 kU_A_/L) peanut‐specific IgE levels at screening, suggesting a relationship between screening IgE and treatment refractoriness; an additional 6 PTAH‐treated participants had screening peanut‐specific IgE levels >100 kU_A_/L and continued in the study.^22^ However, in the present study, the failure to detect a relationship between screening IgE and maximum tolerated dose of peanut protein or symptom severity suggests that biomarkers are currently not a viable replacement for food challenges in determining tolerability to peanut, including that in response to peanut OIT or in identifying threshold levels in response to a food challenge.

In the present study, all of the specific IgE and IgG4 components examined, with the exception of Ara h 8, increased following PTAH treatment at the end of updosing (all *p* < 0.001). Of note, only 27% of PTAH‐treated participants included in this analysis had a positive IgE to Ara h 8, which may affect the sensitivity to detect change. Other studies have examined changes in the immunologic profile over time with OIT and/or reversal of those changes after cessation of OIT or outgrowing allergy.^11^, ^12^, ^13^, ^14^, ^30^, ^31^

Increases in specific IgE and IgG4 levels at the end of updosing and the exit visits were larger in responders (ie, PTAH‐treated participants who tolerated certain doses of peanut protein) than in nonresponders of PTAH treatment. Importantly, the observation that the ratio of IgE or IgG4 levels after updosing and at exit to IgE or IgG4 levels at screening for almost all peanut allergen components for participants in the PTAH group were statistically significant compared with constant levels for placebo indicates that PTAH contains all of the relevant, immunodominant peanut allergens. Whether this indicates that changes in specific IgE and IgG4 levels can be used as a predictor for treatment response is not possible to deduce from the available data. Lack of correlation between specific IgG4 changes and response to other allergen immunotherapy or baseline specific IgE was noted in a small study.^15^ Further evaluation may be needed to identify relationships in changes in IgE and IgG4 levels and response to treatment.

Several important limitations should be noted. Although some analyses were prespecified, some of the analyses were performed post hoc. The study population may not be representative of the broader population of people with peanut allergy because eligibility for the trial required a reaction with ≤100 mg of peanut protein. The 100‐mg dose level has been computationally estimated as the ED_50_—that is, the amount of peanut exposure sufficient to trigger a reaction in approximately 50% of the peanut‐allergic population.^32^ As a result, the data summarized in this manuscript cannot estimate the ability of screening IgE data to predict reactive thresholds nor any other endpoint in the other half of the peanut‐allergic population. Analyses were limited to participants from the US (the majority) and UK. It has been previously published that in different regions of the world, peanut‐allergic patients might be sensitized to different peanut allergens. Although the trial recruited patients from different regions to account for that variability, due to GDPR, the sera of most of the countries were not able to be included in this analysis. Additionally, the ability to detect changes in some parameters might be limited by an inadequately powered sample size; however, this was an exploratory analysis that required additional steps including re‐consenting participants and resubmission for regulatory review and approval, which may have affected sample selection and size. The number of participants with positive screening IgE level was low for some peanut allergen components (ie, Ara h 8 and Ara h 9); therefore, interpretations of data for these allergen components are limited. This limitation may similarly apply to positive screening IgG4 levels for all peanut allergen components. Factors that may have influenced IgE and IgG4 levels at screening, including age, time of last exposure, and previous reactions to peanut, were not considered in these analyses. Nonresponders included both participants who completed the exit DBPCFC but did not tolerate a certain dose, and participants who discontinued prior to the exit DBPCFC. The immunological profile (especially postscreening) may differ between nonresponder completers compared with nonresponder noncompleters, but these subsets were pooled for simplicity. Finally, there are no long‐term data; time points beyond 6 months of maintenance therapy were not examined.

In conclusion, despite the limitations of this explorative evaluation, these results indicate that specific IgE and IgG4 levels at screening are not correlated with screening or exit DBPCFC results, and are not predictive of clinical response to PTAH. Changes in specific IgE and IgG4 levels to all relevant allergens were statistically significant in response to PTAH treatment, signifying that PTAH contains those allergens.

## AUTHOR CONTRIBUTIONS

**Caroline Nilsson:** Conceptualization, Investigation, and Writing – review & editing. **Andrea Vereda:** Project Administration, Investigation, and Writing – review & editing. **Magnus P. Borres:** Conceptualization, Investigation, and Writing – review & editing. **Mats Andersson:** Formal analysis; Writing – review & editing. **Eva Södergren:** Project administration; Writing – review & editing. **Magnus Rudengren:** Conceptualization, formal analysis, and writing – review & editing. **Alex Smith:** Conceptualization, data curation, formal analysis, and writing – review & editing. **Reyna J. Simon:** Conceptualization, Project administration, and Writing – review & editing. **Robert Ryan:** Project Administration; Investigation; Writing – review & editing. **Montserrat Fernández‐Rivas:** Investigation and Writing – review & editing. **Daniel Adelman:** Conceptualization, Investigation, and Writing – review & editing. **Brian P. Vickery:** Investigation; Writing – review & editing.

## CONFLICT OF INTEREST STATEMENT

**Caroline Nilsson:** Grants to my institution and advisory board fees from Aimmune Therapeutics, a Nestlé Health Science company; lecture fees from ALK, GSK, MEDA, and Thermo Fisher; advisory board fees from Novartis. **Andrea Vereda:** Employee of Aimmune Therapeutics, a Nestlé Health Science company. **Magnus P. Borres:** Employee of Thermo Fisher Scientific. **Mats Andersson:** Employee of Thermo Fisher Scientific. **Eva Södergren:** Employee of Thermo Fisher Scientific. **Magnus Rudengren:** Employee of Thermo Fisher Scientific. **Alex Smith:** Former employee and shareholder of Aimmune Therapeutics, a Nestlé Health Science company. **Reyna J. Simon:** Former employee and shareholder of Aimmune Therapeutics, a Nestlé Health Science company. **Robert Ryan:** Employee of Aimmune Therapeutics, a Nestlé Health Science company. **Montserrat Fernández‐Rivas:** Grants to my institution, consulting fees, and payments or honoraria from Aimmune Therapeutics, a Nestlé Health Science company; consulting fees from Novartis, Reacta Healthcare, and SPRIM; payments or honoraria from ALK, Diater, Ediciones Mayo S.A., EPG Health, GSK, HAL Allergy, and Novartis; participation on a data safety monitoring board for DBV Technologies. **Daniel Adelman:** Former employee and shareholder of Aimmune Therapeutics, a Nestlé Health Science company. **Brian P. Vickery:** Grants to my institution from Aimmune Therapeutics, a Nestlé Health Science company, DBV Technologies, FARE, Genentech, NIH‐NIAID, and Regeneron; consulting fees from Aimmune Therapeutics, a Nestlé Health Science company, AllerGenis, Aravax, DBV Technologies, FARE, Reacta, and Regeneron. Former employee and former shareholder owner of Aimmune Therapeutics, a Nestlé Health Science company.

## INFORMED CONSENT STATEMENT

Informed consent or assent, as appropriate, was obtained from all study participants.

## Supporting information

Supplementary Material

## Data Availability

Clarification requests regarding the manuscript and its data can be made to the corresponding author.
