# Supplementary material for: Exploratory immunogenicity outcomes of peanut oral immunotherapy: Findings from the PALISADE trial
Source: Clin Transl Allergy. 2024 Jan 17;14(1):e12326. doi: 10.1002/clt2.12326 (PMC10793676; doi:10.1002/clt2.12326)
Supplement: Supplementary file 1 — Supplementary Material [file CLT2-14-e12326-s001.docx]

# SUPPORTING INFORMATION

# SUPPLEMENTARY TABLES

## Supplementary Table 1. Screening demographic and clinical characteristics of participants included in analyses

| **Characteristic** | **PTAH**  **(n=202)** | **Placebo**  **(n=67)** |
| --- | --- | --- |
| Country, n (%) |  |  |
| United States | 190 (94.1) | 61 (91.0) |
| United Kingdom | 12 (5.9) | 6 (9.0) |
| Age group, n (%) |  |  |
| 4–17 y | 184 (91.1) | 66 (98.5) |
| 18–55 y | 18 (8.9) | 1 (1.5) |
| Screening IgE level, median (IQR) |  |  |
| Peanut-specific IgE, kU_A_/L | 76.89 (24.85, 197.88) | 111.94 (38.42, 364.72) |
| Ara h 1, kU_A_/L | 18.38 (2.98, 52.70) | 35.76 (6.57, 115.10) |
| Ara h 2, kU_A_/L | 38.09 (10.81, 93.87) | 57.37 (21.83, 170.44) |
| Ara h 3, kU_A_/L | 3.14 (0.34, 12.80) | 6.99 (0.38, 37.78) |
| Ara h 6, kU_A_/L | 25.97 (9.30, 66.70) | 27.59 (13.61, 83.48) |
| Ara h 8, kU_A_/L | 0.05 (0.05, 0.12) | 0.05 (0.05, 0.05) |
| Ara h 9, kU_A_/L | 0.05 (0.05, 0.05) | 0.05 (0.05, 0.05) |
| Positive screening IgE level, n (%)^a^ |  |  |
| Peanut-specific IgE | 200 (99.5) | 67 (100) |
| Ara h 1 | 179 (89.1) | 59 (88.1) |
| Ara h 2 | 199 (99.0) | 67 (100) |
| Ara h 3 | 179 (89.1) | 61 (91.0) |
| Ara h 6 | 199 (99.0) | 66 (98.5) |
| Ara h 8 | 54 (26.9) | 15 (22.4) |
| Ara h 9 | 35 (17.4) | 12 (17.9) |
| Screening IgG4 level, median (IQR) |  |  |
| Peanut-specific IgG4, mg_A_/L | 0.58 (0.33, 1.60) | 0.60 (0.34, 1.57) |
| Ara h 1, mg_A_/L | 0.15 (0.15, 0.15) | 0.15 (0.15, 0.32) |
| Ara h 2, mg_A_/L | 0.15 (0.15, 0.15) | 0.15 (0.15, 0.15) |
| Ara h 3, mg_A_/L | 0.15 (0.15, 0.15) | 0.15 (0.15, 0.32) |
| Ara h 6, mg_A_/L | 0.15 (0.15, 0.15) | 0.15 (0.15, 0.23) |
| Ara h 8, mg_A_/L | 0.15 (0.15, 0.15) | 0.15 (0.15, 0.15) |
| Ara h 9, mg_A_/L | 0.15 (0.15, 0.35) | 0.15 (0.15, 0.15) |
| Positive screening IgG4 level, n (%)^a^ |  |  |
| Peanut-specific IgG4 | 155 (77.1) | 52 (77.6) |
| Ara h 1 | 31 (15.4) | 20 (29.9) |
| Ara h 2 | 47 (23.4) | 16 (23.9) |
| Ara h 3 | 42 (20.9) | 21 (31.3) |
| Ara h 6 | 47 (23.4) | 17 (25.4) |
| Ara h 8 | 13 (6.5) | 4 (6.0) |
| Ara h 9 | 56 (27.9) | 16 (23.9) |
| Highest tolerated peanut protein dose during screening DBPCFC, n (%) |  |  |
| 0.3 mg^b^ | 18 (8.9) | 3 (4.5) |
| 1 mg | 16 (7.9) | 6 (9.0) |
| 3 mg | 41 (20.3) | 16 (23.9) |
| 10 mg | 57 (28.2) | 14 (20.9) |
| 30 mg | 70 (34.7) | 28 (41.8) |

^a^For IgE, a value was considered “positive” if ≥0.1 kU_A_/L. For IgG4, a value was considered “positive” if ≥0.3 mg_A_/L.

^b^Participants that could not tolerate any dose level in the screening DBPCFC were assigned a maximum tolerated dose of 0.3 mg.

Abbreviations: DBPCFC, double-blind, placebo-controlled food challenge; IgE, immunoglobulin E; IgG4, immunoglobulin G4; IQR, interquartile range; PTAH, peanut (*Arachis hypogaea*) allergen powder-dnfp.

## Supplementary Table 2. Classification error of maximum tolerated peanut protein dose at screening DBPCFC with screening IgE and IgG4 levels as explanatory variables

| **Responders** | **Dose of peanut protein at screening DBPCFC^a^** | | | | |
| --- | --- | --- | --- | --- | --- |
|  | **0.3 mg^b^** | **1 mg** | **3 mg** | **10 mg** | **30 mg** |
| Observed, n | 21 | 22 | 57 | 70 | 98 |
| Predicted, n | 4 | 4 | 13 | 15 | 26 |
| Classification error, % | 81 | 82 | 77 | 79 | 73 |

^a^Individual participants were only counted under their maximum tolerated dose.

^b^Participants that could not tolerate any dose level in the screening DBPCFC were assigned a maximum tolerated dose of 0.3 mg.

Abbreviations: DBPCFC, double-blind, placebo-controlled food challenge; IgE, immunoglobulin E; IgG4, immunoglobulin G4.

## Supplementary Table 3. Responder rate at exit among participants included in immunoglobulin analyses (N=269)

| **Treatment group** | **300 mg**  **n/N (%)** | **600 mg**  **n/N (%)** | **1000 mg**  **n/N (%)** |
| --- | --- | --- | --- |
| PTAH^a^ | 151/202 (74.8) | 133/202 (65.8) | 97/202 (48.0) |
| Placebo | 5/67 (7.5) | 2/67 (3.0) | 1/67 (1.5) |

^a^One participant in the PTAH group had missing IgE at screening and is not displayed in **Figure 3**.

Abbreviations: IgE, immunoglobulin E; PTAH, peanut (*Arachis hypogaea*) allergen powder-dnfp.

## Supplementary Table 4. True response versus predicted response from a random forest model with screening IgE and IgG4 levels as explanatory variables, separately for each of the peanut protein doses at exit DBPCFC

|  | **Dose of peanut protein at exit DBPCFC^a^** | | | | | |
| --- | --- | --- | --- | --- | --- | --- |
|  | 300 mg | | 600 mg | | 1000 mg | |
| Predicted, n | Yes | No | Yes | No | Yes | No |
| Yes | 82 | 24 | 70 | 34 | 45 | 44 |
| No | 68 | 27 | 62 | 35 | 51 | 61 |
| Error (%) | 45 | 47 | 47 | 49 | 53 | 42 |

^a^Individual participants were counted in analyses of all peanut protein doses they tolerated (ie, participants with response to 1000 mg peanut protein were also responders to 300 mg and 600 mg and were counted in all 3 analyses).

Abbreviations: DBPCFC, double-blind, placebo-controlled food challenge; IgE, immunoglobulin E; IgG4, immunoglobulin G4.

**SUPPLEMENTARY FIGURES**

## Supplementary Figure 1. PALISADE study design.


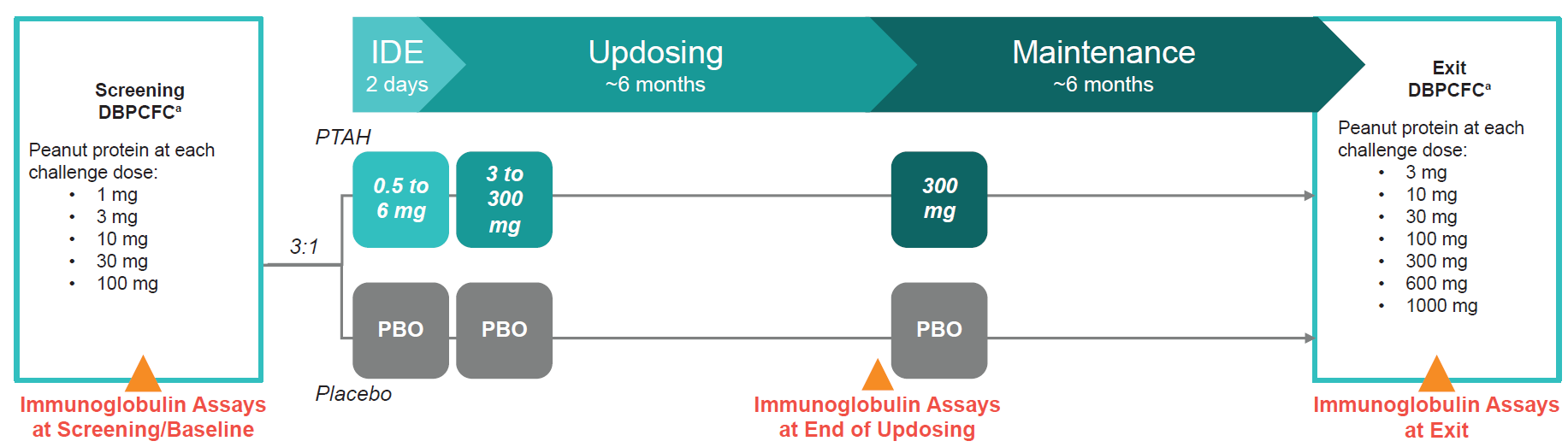


^a^Blood for peanut-specific IgE and IgG4 drawn before DBPCFC.

Abbreviations: DBPCFC, double-blind, placebo-controlled food challenge; IDE, initial dose escalation; IgE, immunoglobulin E; PBO, placebo; PTAH, peanut (*Arachis hypogaea*) allergen powder-dnfp.

## Supplementary Figure 2. Screening IgG4 levels by randomized treatment and maximum symptom severity during the exit DBPCFC.


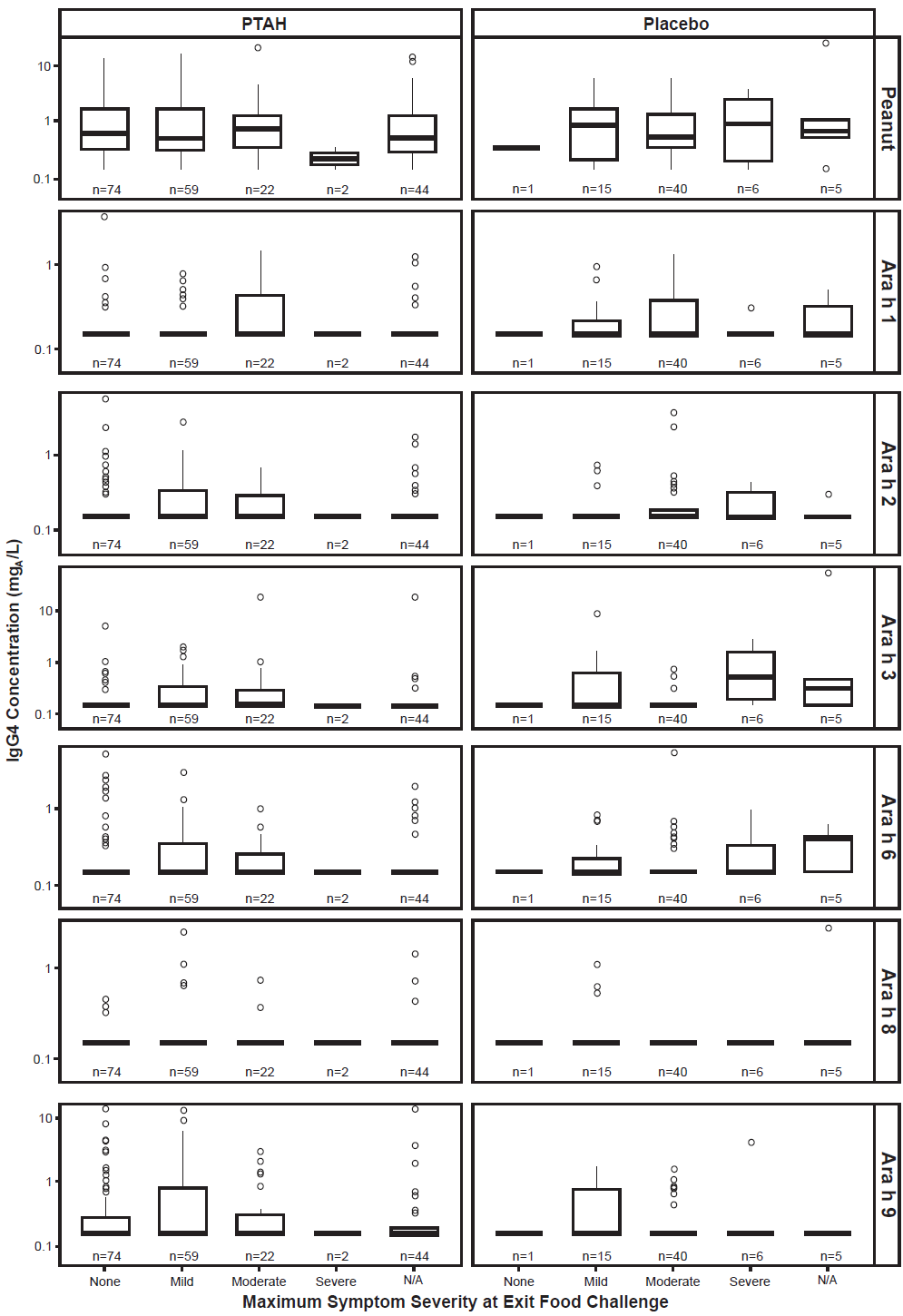


N/A corresponds to participants who did not perform the exit DBPCFC.

Abbreviations: DBPCFC, double-blind, placebo-controlled food challenge; IgG4, immunoglobulin G4; N/A, not applicable.

## Supplementary Figure 3. Geometric mean ratio versus screening in IgE (A) and IgG4 (B) levels by randomized treatment and tolerated peanut protein concentration during the exit DBPCFC (300, 600, or 1000 mg).^a-c^

**A**

**B**

^a^Responders were participants tolerating the specified peanut protein dose during exit DBPCFC with no more than mild symptoms.

^b^Participants who tolerated 600 mg peanut protein also tolerated 300 mg.

^c^Participants who tolerated 1000 mg peanut protein also tolerated 600 mg and 300 mg.

Abbreviations: DBPCFC, double-blind, placebo-controlled food challenge; IgE, immunoglobulin E; IgG4, immunoglobulin G4; PTAH, peanut (*Arachis hypogaea*) allergen powder-dnfp.
